# Virtual versus paper-based PBL in a pulmonology course for medical undergraduates

**DOI:** 10.1186/s12909-023-04421-y

**Published:** 2023-06-13

**Authors:** Heba H. Abo Elnaga, Manal Basyouni Ahmed, Marwa Saad Fathi, Sanaa Eissa

**Affiliations:** 1 Department of Pulmonary, Faculty of Medicine, October 6 University, 28C, Opera City Compound, Sheikh Zayed Giza, Egypt; 2 Department of Medical Biochemistry and Molecular Biology, Faculty of Medicine, Ain Shams University, Cairo, Egypt; 3 Medical Education Department, Faculty of Medicine, Ain Shams University, Cairo, Egypt; 4 Department of Medical Microbiology and Immunology, Faculty of Medicine, Ain Shams University, Cairo, Egypt

**Keywords:** Problem-based learning, Virtual patient, Medical education

## Abstract

**Background:**

Problem-based learning (PBL) remains a valid and effective tool for small-group medical education. Using Virtual patients (VP) case simulation in PBL is a recognizable educational method that has successfully prepared students to focus learning on core information that uses realistic patient-based cases relating to everyday clinical scenarios. Using other modalities as the virtual patient in PBL instead of the paper-based methods remains debatable.

This study aimed to evaluate the effectiveness of using VP case simulation mannequin in PBL versus the PBL in paper-based cases in improving the cognitive skills by comparing the grades of a multiple-choice question test and assess its ability to reach students' satisfaction using questionnaire with Likert survey instrument.

**Methods:**

The study was conducted on 459 fourth-year medical students studying in the pulmonology module of the internal medicine course, Faculty of Medicine, October 6 University. All students were divided into 16 PBL classes and randomly divided into groups A and B by simple manual randomization. The groups were parallel with a controlled cross-over study between paper-based and virtual patient PBL.

**Results:**

The pre-test showed no significant difference between both, while post-test scores were significantly higher in both VP PBL cases 1 discussing COPD (6.25 ± 0.875) and case 2 discussing pneumonia (6.56 ± 1.396) compared to paper-based PBL (5.29 ± 1.166, 5.57 ± SD1.388, respectively) at *p* < 0.1 When students in Group A experienced PBL using VP in case 2 after paper-based PBL in case 1, their post-test score improved significantly. (from 5.26 to 6.56, *p* < .01). Meanwhile, there was a significant regression in the post-test score of the students in Group B when they experienced the paper-based PBL session in case 2 after using PBL using VP in case 1, (from 6.26 to 5.57, *p* < .01). Most of the students recommended using VP in PBL as they found VP was more engaging and inducing concentration in gathering the information needed to characterize the patient’s problem than in a classroom- paper-based cases session. They also enjoyed the teaching of the instructor and found it a suitable learning style for them.

**Conclusion:**

Implementing virtual patients in PBL increased knowledge acquisition and understanding in medical students and was more motivating for students than paper based PBL to gather the needed information.

**Supplementary Information:**

The online version contains supplementary material available at 10.1186/s12909-023-04421-y.

## Background

The importance of the educational environment in medical schools has attracted the attention of educational institutes. Students' study significantly impacts their course satisfaction, sense of well-being, aspirations, and knowledge acquisition [1–8]. Around fifty years ago, collaborative learning through case-based or PBL scenarios has been a brilliant way to gain and develop workplace knowledge associated with specific competencies [9].

The process of PBL with numerous variants of the techniques craft the students to detect their knowledge's limitations; they detect learning gaps essential to answer questions deficient in their reserved knowledge [3].

A problem is generally provided to students on paper or in digital format, and it is then studied and discussed in small groups across two or three sessions. This mode of delivery, however, lacks the realism of a patient encounter; it does not develop or assess students' behavioural talents, reasoning, decision making, or communication skills [10].

Virtual patients are interactive computer simulations of real-life clinical scenarios used for training, education, or assessment in the health professions [11]. This includes various systems that employ diverse technologies and satisfy varied learning requirements [12]. The student is placed in the role of a health care professional, making judgments concerning the type and order of clinical information gathered, differential diagnosis, and patient management and follow-up [8]. It is predicted that virtual patients will largely address clinical reasoning learning demands [13, 14]. However, the impact of using virtual patients on other educational outcomes has not been thoroughly investigated [12, 15]. Some literature reported that the educational use of virtual patients as one of the techniques for applying PBL provided a successful learning tool where virtual patients provide learners with simulated healthcare experiences while also providing methods for information collecting and clinical decision-making in the case scenario [16].

Another study confirmed that medical students need to be exposed to simulation education experiences on a regular basis in order to maintain psychological stability and provide competent medical care in a clinical setting [17].

Several studies have discovered that simulation can be transformed into a powerful strategy for appropriately training health professionals to effectively address today's changing world's challenges [18].

Furthermore, a virtual patient platform in conjunction with a diagnostic reasoning framework could be used for education, diagnostic assessment, and improved correct diagnosis [19].

When comparing PBL using paper-based, and virtual patients, they concluded that employing virtual patients can more effectively increase abilities and at least as effectively improve knowledge. Clinical reasoning, procedural skills, and a combination of procedural and team skills improved; proof of effectiveness from various income countries indicates virtual patients' global applicability. Further research should be conducted to investigate the the potential positive effects of VPL on clinical reasoning that might be gained by introducing such learning support [20, 21].

Many countries are beginning to develop simulation and recent innovations in medical education for both undergraduates and postgraduates, with the hope of supporting and improving patient care delivery [22].

The use of VP mannequins is expected to progressively replace the present less genuine paper-based versions of PBL, presuming that future generations are looking forward to more creative learning methods [16].

Using other modalities as the virtual patient in PBL instead of the paper-based methods remains debatable.

This study aimed to evaluate the the potential positive effects of using VP case simulation mannequin in PBL versus the teaching modalities of PBL in paper-based cases in improving the cognitive skills by comparing the grades of a multiple-choice question test and assess its ability to reach students' satisfaction using questionnaire with Likert survey instrument.

## Methods

### Study design

This is a single-center, randomized, parallel-group with a controlled cross-over study design conducted on 459 fourth-year medical students, 279 males (60.8%) and 180 females (39.2%), in the pulmonology module of internal medicine course, Faculty of Medicine, October 6 University.

The research was conducted as part of a pre-existing problem-based learning curriculum. Students enrolled in the course voluntarily participated. All study activities, including the completion of multiple-choice self-assessment questions to evaluate learning, were completed within the framework of the course, in accordance with the guidelines and legal regulations of the faculty of medicine, October 6 University without extracurricular activities required.

As is customary for all courses at our medical school, all fourth-year medical students were divided into 16 PBL classes at the start of the module. In our study, 8 of which were randomly assigned to group A (233 students) and 8 of which were assigned to group B (226 students) by simple manual randomization method [23].

**Fig. 1:**
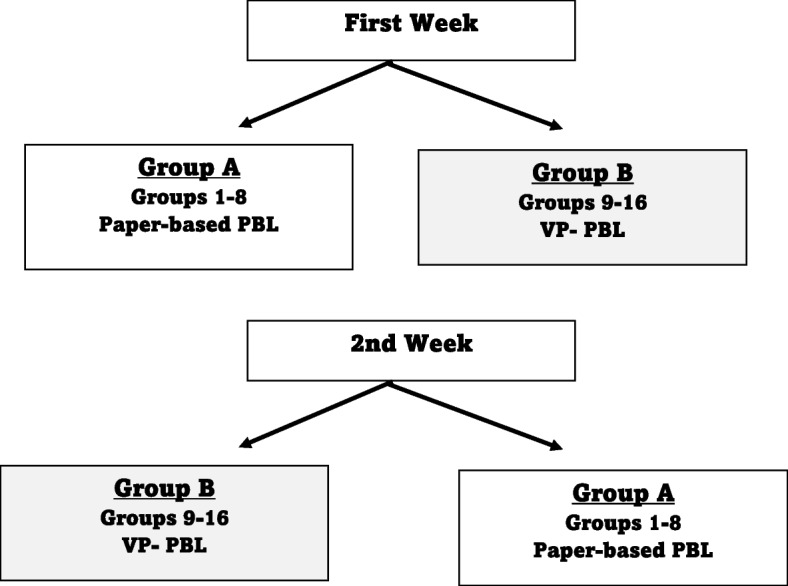
Flow chart of participant selection

The PBL curriculum required all groups to attend a two-hour meeting each week and another 2 h session for discussion and performing the posttest. During the first week of the module, In a paper-based PBL format, Group A faced structured teaching objectives related to managing chronic obstructive pulmonary disease (COPD). Meanwhile, Group B conducted the same COPD case using the virtual patient simulator by interviewing a mannequin that delivered information that matched the written script [24], Fig. 1.

The students were crossed over to the opposite modality in the second week. During this week, Group A encountered structured teaching points related to the management of a case of pneumonia by the virtual patient, interviewing the mannequin simulator, while Group B completed the same case in a paper-based PBL with identical teaching points.

The study's virtual patient mannequin, ALEX-PCS, is a high-fidelity manikin patient communication simulator that incorporates the most recent computer hardware technology, similar to the SimMan. This wireless high-fidelity manikin has been programmed to provide an extremely realistic full-body patient presentation. It also achieves the highest level of realism and provides a diverse set of high stakes learning scenarios.

Cases were developed from real clinical records collected locally. Their content was matched for topic and difficulty level and set up to be as similar as possible in terms of complexity and case type. All Students participated in other similar activities throughout the pulmonology curriculum as lectures, small group teaching, and skill lab. All PBL faculty facilitators involved in teaching either the paper-based PBL or using the virtual patient mannequinare underwent training workshops and were given particular instructions to reinforce the teaching points related to managing the assigned PBL cases in both groups.

The study was approved by the ethical committee of the Faculty of Medicine of both October 6 University and Ain Shams University (ethical approval number: MS 769/ 2021). Informed, written consent was obtained from all participants of the study. All methods were performed in accordance with the legal regulations of the faculty of medicine, October 6 University without extracurricular activities.

### Assessment methods

Each student completed an online multiple-choice question and submitted it successfully as a pre-experience and post-experience test specific to the topic area. The tests covered the pulmonology case PBL session either by using virtual patient case simulation PBL or paper-based teaching modalities of PBL both pre-experience and post-experience tests assess the learning of the teaching points and students' application of knowledge.Both pre-experience and post-experience tests were drawn from the question bank of the faculty of medicine, October 6 University which was previously used in different exams. they were tested before for their validity and reliability by the expert members of faculty of medicine according to the results of facility and discrimination indices and the presence of functional distractors of each question calculated by the software program.

Student performance on pre- and post-experience assessment tests had no bearing on their final course grade. Both pre-tests and post-tests were scored using a web-based course management system using an answer key written prior to administration, and each student received an individual score [25].

At the end of the module, all students were asked to complete a questionnaire using Likert survey instrument with response options ranging from 0 = strongly disagree to 4 = strongly agree) to reflect their experience and satisfaction with the virtual patient case simulation in Problem-based learning (PBL) compared to paper-based teaching modalities of PBL of a written case scenario [26].

The structured self-administered questionnaire was translated into Arabic to make it simple and understandable for all participants. We contacted the students via the Microsoft Teams application. We invited them to participate in the study via an electronic link with a questionnaire after explaining the purpose of this research.

The questionnaires were designed following thorough research of related literature. They were authorized by Ain Shams University's specialist staff members in public health and community medicine, who reviewed their validity and reliability [27, 28].

The questionnaire included the students' socio-demographic characteristics involving age, sex, and nationality. Moreover, the consent, which feeds agreeing of the student to participate in the study and to answer the questionnaire with the researcher's contacts, was comprised.

### Inclusion criteria

This randomized, controlled cross-over study included all undergraduate fourth-year medical students enrolled in the Internal Medicine course in the 2021–2022 academic year of the pulmonology course at the faculty of medicine, October 6 University. The pulmonology module is an integral component of the internal medicine course's curriculum, lasting four weeks and consisting of an average of eight lectures per week, small group learning and discussion sessions (centered on solving clinical cases), and others for demonstration and teaching clinical examination of real patients.

All the students enrolled in the study had equivalent knowledge and skills in pre-clinical sciences, and their IT skills were comparable. Therefore, no assessable differences could be observed between the included students at the study's baseline.

### Exclusion criteria

• Students who were not registered in the chest diseases module.
• Students who failed previously in the module and are repeating it,
• Students who did not regularly attend the teaching sessions of PBL or simulation by virtual patient.

### Data analysis

The average score for each of the pre-test and the post-test each week were calculated and compared between the two groups of the paper-based PBL and Virtual patient group for both the COPD and pneumonia cases.

Comparisons between the two groups were made using independent t-tests, and comparisons within the same group pre and post-tests were made using paired t-tests.

Analysis was performed using statistical software (SPSS version 23; SPSS, Inc., Chicago, IL). Data are presented as mean ± SD for quantitative data and frequency (%) for categorical data; a *p*-value of < 0.05 was considered statistically significant.

## Results

 The study included 459 students studying in the Respiratory module of internal medicine course in grade 4, Faculty of Medicine, October 6 University. The participants were 279 males and 180 females, with a mean age of 20.81 ± 2.6 years (Table 1).

**Table 1:** Characteristic data of participating students

Age (years)	Mean ± SD	
	20.81 ± 2.601	
Gender	Frequency (n.)	Percent (%)
Female	180	39.2
Male	279	60.8
Nationality	Frequency (n.)	Percent (%)
Egypt	421	91.7
Jordan	14	3.1
Nigeria	10	2.2
Yemen	8	1.7
Botswana	2	0.4
Sweden	2	0.4
Lebanon	2	0.4
Total	459	100.0

The participants studied two different case scenarios. During the study on each case, a pre-test was done for all students, and then the students were divided into two groups; Group A, where paper-based learning (PBL) was implemented, and Group B, where virtual-patient learning (VP) was implemented after which a cross-over rearrangement was done for the second case scenario. A post-test was done after studying each case.

In both cases, after conducting PBL by either paper-based or virtual patient approach, the post-tests students' scores were significantly higher than their pre-tests' scores; *p* < 0.0001 (Table 2). There was no significant difference between groups A and B regarding the total pre-test scores (*p* > 0.05). At the same time, the post-test total scores are significantly increased in the virtual PBL group (group B in case 1 and group A in case 2); *p* < 0.01 (Table 3).

**Table 2:** Comparison between Pre and Posttest mean scores of the students in group A and the students in group B in both case1 and case 2

		Group A		Group B	
		Mean ± SD	Significance	Mean ± SD	Significance
Case 1	Pre-test	4.30 ± 1.450	T = -8.774 *P* < 0.0001	4.39 ± 1.402	T = -17.437 *P* < 0.0001
	Post-test	5.26 ± 1.154		6.26 ± 0.872	
Case 2	Pre-test	3.55 ± 1.338	T = -21.253 *P* < 0.0001	3.50 ± 1.351	T = -22.181 *P* < 0.0001
	Post-test	6.56 ± 1.396		5.57 ± 1.388	

Data are presented as Mean ± SD; *p*-value < 0.05 is statistically significant

Group A: who experienced paper-based PBL in case 1 and PBL using VP in case 2

Group B: who experienced PBL using VP in case 1 and paper-based PBL in case 2

**Table 3:** Comparison between group A and group B mean total scores after Pre and Post-test in both Case 1 and case 2

		Group A	Group B	Significance
		Mean ± SD	Mean ± SD	
Case 1	Pre-test	4.30 ± 1.450	4.39 ± 1.402	T = -0.650 *P* = 0.516
	Post-test	5.26 ± 1.154	6.26 ± 0.872	T = -9.106 *P* < 0.01
Case 2	Pre-test	3.55 ± 1.338	3.50 ± 1.351	T = 0.321 *P* = 0.748
	Post-test	6.56 ± 1.396	5.57 ± 1.388	T = 6.849 *P* < 0.01

Data are presented as Mean ± SD; *p*-value < 0.05 is statistically significant

Group A: who experienced paper-based PBL in case 1 and PBL using VP in case 2

Group B: who experienced PBL using VP in case 1 and paper-based PBL in case 2

Comparing the scores of post-tests between case 1 and case 2. There was a significantly higher score in case 2 in Group A (from 5.26 to 6.56, *p* < 0.01). Meanwhile, the score was significantly decreased in Group B (from 6.26 to 5.57, *p* < 0.01) (Table 4).

**Table 4:** Comparison between mean post-test results of students in groups A and B when they experienced the paper-based PBL versus PBL by VP in both cases

	Post-test paper-based PBL	Post-test PBL-VP	p
	Mean ± SD	Mean ± SD	
Group A	5.26 ± 1.154	6.56 ± 1.396	< 0.0001
Group B	5.57 ± 1.388	6.26 ± .872	< 0.0001

Data are presented as Mean ± SD; *p*-value < 0.05 is statistically significant

Group A: who experienced paper-based PBL in case 1 and PBL using VP in case 2

Group B: who experienced PBL using VP in case 1 and paper-based PBL in case 2

Survey.

A survey questionnaire reflecting the students' experience and reach of their satisfaction with the (PBL) using VP simulation compared to paper-based teaching modalities of PBL of a written case scenario. The student's responses to the survey were distributed on a Likert scale score of 0–4.

**Fig. 2:**
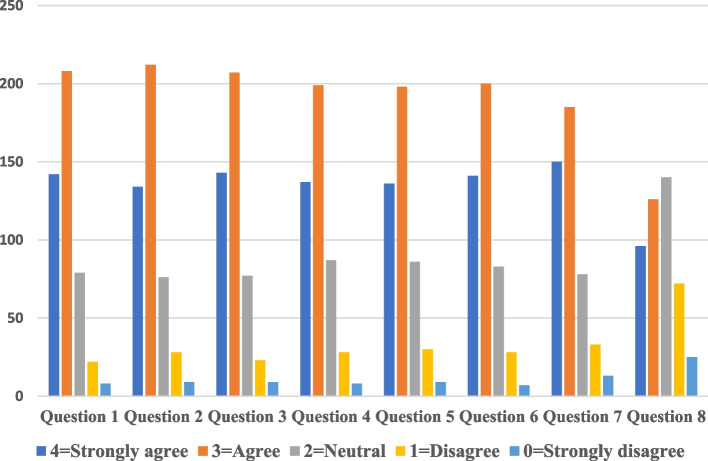
The responses to various questions differ significantly.(*p* < 0.01). Likert scale of questions 1 to 7: 0 = strongly disagree, 1 = Disagree, 2 = Neutral, 3 = Agree, 4 = strongly agree. Likert scale of question 8 is 0 = poor, 1 = Neutral, 2 = Good, 3 = Very good, 4 = Excellent

There are significant differences in the responses to various questions (*p* < 0.01) (Fig. 2).

Regarding question 1, 142 (30.9%) students strongly agreed, and 208 (45.32%) students agreed that the teaching method of the case by the virtual patient was more helpful and effective than a classroom- paper-based case session.

While in question 2, 134 (29.2%) students strongly agreed, and 212 (46.19%) students agreed that the teaching method of the case by the virtual patient provided them with learning materials and activities to promote seeking knowledge more than a classroom- paper-based cases.

By asking whether the students enjoyed teaching the case by the virtual patient more than a classroom- paper-based cases session in question 3, 143 (31.2%) students strongly agreed, and 207 (45.1%) students agreed.

Concerning question 4,137 (29%) students strongly agreed, and 199 (43.35%) students that the teaching method of the case by the virtual patient was motivating and helped them to learn more than a classroom- paper-based cases session.

Considering question 5, 136 (29.6%) students strongly agreed, and 198 (43.13%) students agreed that the teaching method of the case by the virtual patient was engaging and seemed to reinforce the students effectively to introduce new knowledge they needed, to characterize the patient's problem more than a classroom—paper-based cases session.

Concerning question (6), 141 (30.7%) students strongly agreed, and 200 (43.57%) students agreed that the way their instructor(s) taught using the virtual patient suited their learning style more than a classroom- paper-based cases session.

Regarding the recommendation of the students to use VP in PBL in upcoming teaching sessions (question 7), 150 (32.7%) students strongly agreed, and 185 (40.31%) agreed, while only 33 (7.2%) students disagreed and 13 (2.8%) strongly disagreed.

Finally, question 8 answer adopted a different theme where 140 (30.50%) students rated the quality of the teaching session with the virtual patient as "Good," 126 (27.5%) as very good, and 96 (20.9%) as excellent, in comparison to the classroom- paper-based cases session.

A discussion was made by a focal group of faculty members, who are the practitioners of both PBL teaching methods.Overall, they provided positive and encouraging responses that virtual PBL method for teaching pulmonary cases was an enjoyable experience. However, we could not make conclusions about this issue due to the small number of staff participating in this study.

## Discussion

PBL is abundantly used in advanced medical education; in 2005, over 70% of medical schools reported employing PBL in some small group teaching for medical students in the pre-clinical years [29]. However, despite the importance of PBL as a pedagogical method of learning, in improving students' abilities such as clinical reasoning, problem-solving and critical thinking [30–32]. The data about the efficacy and outcomes of using other modalities as the virtual patient compared with the paper-based methods remains inadequate [33, 34], especially in Egypt.

After studying each of the 2 cases (COPD and Pneumonia), the chances of the medical students answering the post-test questions correctly after completing the PBL session via virtual patient recorded significantly better scores than their colleagues who completed PBL by the paper-based.

These results also reveal that virtual patient PBL increased post-test performance independent of the week the students received the intervention, This is congruent with the findings of other studies, which found that incorporating digital learning objects in PBL increased cognitive, metacognitive, affective, and total learning processes or outcomes [35] This was evident by comparing the post-test scores between case 1 and case 2 in each group. There was a significantly higher score in case 2 in Group A in which they studied PBL by the virtual patient Meanwhile, the score of post-tests between case 1 and case 2 was significantly decreased in Group B.

Ultimately There was no discernible difference between the pre-test scores of the 2 cases of the two groups of students who participated in the study, which means that both groups of students had an equal level of basic knowledge before they started to study any of the 2 cases.

Given the significant improvement in postexperience test scores after using VP in PBL compared with postexperience test scores after classroom- paper-based PBL sessions, we conclude that the method of teaching by PBL is effective in improving the students' competencies. Meanwhile, using the virtual-patient learning in PBL showed excellence over the paper-based methods in enhancing the students' focus on core information and knowledge relevant to patient case scenarios. According to previous studies, dedicated software can help learners produce explanations, structure exercises, and make them more manageable [36].

We designed this study to compare the application of PBL by both modalities, the paper-based and by the virtual patient with cross-over method, to achieve complete fairness for all students to have the same opportunity to experience interactive small group activities of PBL in both methods that revolve around working through a patient case. It was apparent that the questions in both weeks were matched in their level of difficulty.

The results of this study are consistent with previous findings regarding both paper-based PBL and virtual-patient PBL in clinical-level medical students [37].

The current study verified that the primary advantage of PBL using VP enhanced the students learning, comprehension and recalling of core information relevant to real patient scenarios. This was evidenced by Students' test scores which significantly improved when undertaking VP PBL compared to the paper-based PBL. Previous research indicated that incorporating technology into education provides students with an engaging learning experience, allowing them to stay more engaged in the material without being distracted [38, 39]. When the students in Group B encountered structured teaching points related to the management of a chronic obstructive pulmonary disease (COPD) case 1 by the virtual patient meeting a mannequin, they achieved significantly higher scores in the post-test of their scores than when they crossed over and encountered structured teaching points related to the management of a case of pneumonia by the paper-based PBL format (case 2).

These findings were also reported in group A whose scores on the post-test after encountering structured teaching points related to the management of a case of (COPD) case 1 by the paper-based PBL format improved to be significantly higher than their score in the post-test when they crossed over and encountered structured teaching points related to the management of a case of pneumonia (case 2) by the virtual patient.

In addition, there were non-significant statistical differences between the scores of the pre-test o the 2 cases in the same group, which denotes the equal difficulty of both cases.

This study supports the concept advanced by others that such visual, aural, and tactile cues engage learners beyond the solely cognitive features of standard PBL [40]. Another study proved that students prefer the idea of having a dialogue with a patient in order to better understand that patient”s problem [15].

As a result, VPs, especially those using a mannequin, can be effectively integrated into clinical education by coordinating their use with other learning activities (e.g., didactics, clinical experiences), by making room in the course by eliminating some lectures and textbook assignments, and by taking a voluntary rather than obligatory approach.

It was evident that employing a virtual patient in PBL offers the learner an enjoyable environment to make decisions and understand the ramifications of those actions [41]. Such interaction may strengthen learning concepts beyond a vocal discussion of a textual example, such as VP interactive activities encouraging deeper learning, accentuating understanding and application of knowledge over memory and recall. It was evident in a study that found a general favourable effect from the use of various educational technology in PBL. Making disciplinary thinking and techniques clear; giving a platform to stimulate articulation, cooperation, and reflection; and lowering perceived cognitive load were all positive consequences for student learning.[42].

This was obvious, after analysis of the questionnaire, about the level of satisfaction regarding the use of the virtual patient in PBL at the end of the module where all students had experienced both the written and virtual patient I PBL sessions.

Most students preferred learning by VP PBL to paper-based PBL and agreed that VPs were more efficacious concerning learner satisfaction and learning outcomes.

Limited previous research on the utility of VPs within medical education found that VPs were equally effective compared to other simulation methods [43–45].

The majority of the students either strongly agreed, or agreed that the virtual patient provided them with learning materials and activities which encouraged exploring new knowledge than a classroom- paper-based cases session. These findings are consistent with McLean's who concluded that students consider computer system as the most helpful form of communication and resource delivery for PBL in medicine [46].

This study identified student preferences and agreed with Huwendiek and his colleagues on the usefulness of sequencing and matching VPs with other activities and evaluations. An example of an active learning activity is having students monitor major discoveries presented in a VP mannequin. Creating a summary statement from a VP's history is an example of positive activity. VPs provide the benefit of a uniform case presentation [47].

Our results showed that most of the students enjoyed the instructor's approach of presenting the case by the virtual patient more than a classroom- paper-based cases session, as approved in another study in which educational technology assist students and their facilitators in making disciplinary thought clear [36].

The interactive nature of the e-learning model engages the participant, shifting them to an active learning experience. This removes the didactic passivity of a teaching-centered approach, as previously proved by Ruiz and his coworkers [48, 49].

Concerning the motivation for learning, Most of the students strongly agreed, and students agreed that the teaching method of the case by the virtual patient was motivating and helped them to learn more than a classroom-paper-based cases session. This was supported by the findings of Dornan et al., who contended that information system can create motivation to the students [50].

There was a positive effect of PBL by VPs on engaging the students in a simulation of full-body mannequins with a real-life case scenario that inspired students to gain experience in practice and facilitate high-fidelity learning. Through active engagement and team cooperation, simulation supplies crucial parts of a reflective practitioner's future education. This was clear during measuring student satisfaction where the greatest number, found that the teaching method of the cases by the virtual patient enabled them to characterize the patient's problem more than the paper-based PBL. This goes in accordance with Gesundheit et al. (2009), who found that medical students were highly satisfied with using VPs, which could be a variable in their engagement in learning activities [42, 51].

The results of our study revealed that many students strongly agreed, and agreed that the method their instructor(s) taught the virtual patient was more conducive to their learning style than a classroom-paper-based case session. This student's opinion encourages teachers participating in medical education to seek a modification of the paper-based model of learning and education. This was also recommended by Hmelo-Silver, and his colleagues [36].

However, a review of literature by a fourth-year medical student stated that students did not wish to see paper-based PBL instructor-led instruction but needed enhancement by innovative teaching techniques [52].

This was obvious in the rate of the quality of the teaching session with the virtual patient in comparison to the classroom- paper-based cases session, where most of students rated it an excellent session,, very good, and "Good."

On the other hand, most t students enjoyed how their instructor presented the PBL session using the VP they recommended and favored it as a learning technique in all aspects of the curriculum. This is consistent with the findings of Olaussen and his colleagues study [53].

Moreover, a considerable number of students recommended using virtual patient in upcoming teaching sessions rather than a classroom- paper-based cases session. These findings indicated positive student feedback on using VP in PBL, which met their expectations during the session. The students had a better area for active engagement by using their whole body and all five senses associated with their intellectual, psychological, and interactional skills.

Another study found that VPs as gamification in medical education could successfully inflate skills and effectively improve knowledge, clinical reasoning, procedural skills, and a combination of procedural and team skills [54].

## Limitations

Limited number of cases conducted ( only two cases) in only one module ( the pulmonary module) in one institution in Egypt,.

Also, the completion of the case was dependent on internet connectivity, which could cause the mannequin's response to be delayed during the session and distract the students' concentration.

## Conclusion

The findings of this single-center study demonstrated that using virtual patients mannequins was more effective than paper-based PBL for knowledge acquisition and student’satisfaction in fourth-year medical students during PBL of COPD and pneumonia cases.

## Supplementary Information

**Additional file 1.** 

## Data Availability

The datasets generated during and/or analysed during the current study are available from the corresponding author on reasonable request.

## Abbreviations

PBL: Problem-based learning
VP: Virtual patients
