# Supplementary material for: Virtual versus paper-based PBL in a pulmonology course for medical undergraduates
Source: BMC Med Educ. 2023 Jun 13;23:433. doi: 10.1186/s12909-023-04421-y (PMC10262484; doi:10.1186/s12909-023-04421-y)
Supplement: Supplementary file 1 — Additional file 1. [file 12909_2023_4421_MOESM1_ESM.docx]

Appendix 1:

**Case 1 (score: 7)**

History:

A 69-year-old female retired cigarette smoker male patient, 20 cigarettes per day for 40 years. She presents with a chief complaint of increased shortness of breath and a change in the quantity and color of his sputum over the past week. The sputum is usually scant and clear but during most of the days of the previous ten years. However, recently it has become yellow and continues all day. she has become progressively short of breath over the last five years. She is now dyspneic at rest. She denies asthma, childhood respiratory problems, and allergies.

Physical Examination:

- Obvious respiratory distress with prominent use of accessory muscles.
- Temperature 37.3; Blood pressure 140/90; pulse 75; respiratory rate 28.
- Head/neck reveal distended neck veins throughout expiration.
- Chest reveals an increased A-P diameter; reduced chest wall excursion; lungs equal resonance to percussion; diaphragms low and immobile; auscultation reveals a prolonged expiratory phase with diminished breath sounds and generalized rhonchi.
- The heart reveals heart sounds distant with regular rhythm and no murmurs. With signs of pulmonary hypertension with a loud P2, raised JVP, tachycardia, and hypotension.
- Extremities reveal trace pitting edema of the lower extremities.
- Chest x-ray reveals hyperinflation of lungs with an increase in the retrosternal space; low, flattened diaphragms; hyper-lucent lung fields with a paucity of vascular markings in the periphery but prominent hila and narrow heart silhouette.
- Laboratory reveals WBC 8,500 with normal differential and Hgb 14.7 gm.
- The physician in the OPD referred him to do spirometry, revealing obstructive pattern spirometry (FEV1 1.06L, 36% of predicted, and FEV1/FVC ratio 38%) with no reversibility (FEV1 improvement less than 15% after nebulized salbutamol).

So, the physician prescribed her short-acting inhaled beta-2 agonist on demand and antibiotic amoxicillin/ clavulanic acid 1 gm twice daily, advising him to drink plenty of fluids for follow-up after one week.

One week later, the patient revisited the physician without any improvement in his dyspnea. Although she used his inhalation beta 2 agonists more than ten times per day, but the color of his sputum became whitish.

Therefore, the physician decided to prescribe her a combination of inhaled long-acting beta 2 agonists with inhaled long-acting methacholine antagonists. Moreover, he referred the patient to the physiotherapy department to improve the strength of respiratory muscles.

One month later, the patient showed marked improvement in her dyspnea and cough.

**Objectives:**

1. Define COPD
2. Outline differential diagnosis of COPD
3. List clinical presentation of COPD
4. Interpretate investigations for COPD
5. Outline pharmacological and non-pharmacological treatment of COPD
6. List complications of COPD

**Test on COPD case**

1. COPD (chronic obstructive pulmonary disease) is the same as bronchial asthma.
2. True
3. False
4. What is one of the most prevalent symptoms in patients with COPD, chronic bronchitis?
5. Dry cough
6. Cough with sputum
7. hemoptysis
8. chest pain
9. COPD is almost always caused by ____________.
10. Pollution
11. Dust
12. Smoking
13. Exposure to asbestos
14. What is true about the definition of COPD?
15. It is reversible airway obstruction
16. It is progressive airway obstruction
17. It is triggered by strong odors
18. It has seasonal variation
19. Which of the following is the most likely investigation to diagnose COPD?
20. Chest X-ray
21. Spirometry
22. Bronchoscope
23. Sputum analysis
24. Which of the following is the first-line treatment for COPD?
25. Inhaled corticosteroids
26. Systemic corticosteroids
27. Inhaled beta 2 agonists
28. Antileukotrienes
29. Which of the following is a complication of COPD?
30. Corpulmonale
31. Stridor
32. Lung abscess
33. Toxemia

**Appendix 2:**

**Case 2 pneumonia (score: 7)**

A 61-year-old man presents to the hospital with fever, mostly dry cough with scanty sputum, and difficulty breathing. He also reports feeling very tired and unwell. He has a history of hypertension, which is controlled with enalapril. On exam, his pulse is 120 bpm, his temperature is 101.6°F (38.7°C), and his oxygen saturation is 88%. He appears acutely ill.

He is admitted to the hospital in an isolation room and is started on oxygen, intravenous fluids, and venous thromboembolism prophylaxis. Blood and sputum cultures are ordered.

Chest x-ray shows bilateral lung infiltrates, and computed tomography of the chest reveals multiple bilateral lobular and subsegmental areas of ground-glass opacity of bronchopneumonia.

Laboratory findings showed CBC, WBC count: 4000, with neutrophilia and lymphopenia, ESR and CRP are high

Sputum gram stain showed few pus cells

A nasopharyngeal swab is sent for real-time reverse transcriptase polymerase chain reaction testing, and the result comes back positive for severe acute respiratory syndrome coronavirus 2 (SARS-CoV-2) a few hours later.

**Objectives:**

1. Definition of pneumonia
2. Types of pneumonia
3. Clinical presentation of pneumonia
4. Investigations of pneumonia
5. Complications of pneumonia

**Test:**

1. Which of the following defines pneumonia?
2. acute infection of the lower respiratory tract caused by a wide variety of microorganisms
3. A severe chest cold
4. Advanced bronchitis
5. It is the result of the development of bacterial colonization
6. **Which of the following is most accurate regarding the presentation of patients with pneumonia?**
7. Purulent sputum is a common feature of pneumonia that is caused by atypical pathogens, excluding Legionnaire's disease
8. Bacterial pneumonia has been associated with both hyperthermia and hypothermia, as well as with tachycardia and bradycardia
9. Lobar consolidation is a typical feature of pneumonia caused by a viral infection
10. Dry cough is more commonly associated with pneumonia caused by Streptococcus pneumoniae
11. **Which of the following is recommended in the workup of patients with community-acquired pneumonia CAP?**
12. Sputum Gram stain and culture should be routinely obtained in adults with CAP managed in the outpatient setting
13. Blood cultures should be routinely obtained in adults with CAP managed in the outpatient setting
14. If a pandemic virus is circulating in a community, testing adults with CAP using a rapid viral molecular assay (PCR) is preferred
15. Adults with CAP should routinely undergo urine testing for Legionella antigen
16. Which of the following is **NOT** a common complication of pneumonia?
17. Confusion
18. Renal impairment
19. Shock
20. Respiratory failure
21. Corpulmonale
22. Pleural effusion
23. Which of the following is NOT a type of anatomical classification of pneumonia?
24. Multilobe pneumonia
25. Bronchopneumonia
26. Bacterial pneumonia
27. Segmental pneumonia
28. Which of the following is NOT a type of microbiological classification of pneumonia?
29. Bacterial pneumonia
30. Fungal pneumonia
31. Viral pneumonia
32. Ventilator-associated pneumonia
33. Is the diagnosis of pneumonia confirmed by radiological findings?
34. True
35. False

**Appendix 3: Assessment of students' Satisfaction with PBL using Virtual Patient**

| **Do you agree with the following statements about teaching PBL with VP** | | | | | | |
| --- | --- | --- | --- | --- | --- | --- |
|  | | **Strongly agree4** | **Agree** | **Neutral** | **Disagree** | **Strongly disagree** |
| **The teaching method of the PBL by the virtual patient:** | | | | | | |
| **1** | **It was more helpful and effective than a classroom- paper-based PBL session.** | **4** | **3** | **2** | **1** | **0** |
| **2** | **Provided me with learning materials and activities to promote my learning more than a classroom- paper-based cases session.** | **4** | **3** | **2** | **1** | **0** |
| **4** | **It was motivating and helped me to learn more than a classroom- paper-based cases session** | **4** | **3** | **2** | **1** | **0** |
| **5** | **Was engaging and induced concentration in gathering the information I needed to characterize the patient's problem more than in a classroom- paper-based cases session** | **4** | **3** | **2** | **1** | **0** |
| **Regarding the instructor:** | | | | | | |
| **3** | **I enjoyed how my instructor taught the PBL by the virtual patient more than in a classroom- paper-based cases session.** | **4** | **3** | **2** | **1** | **0** |
| **6** | **The way my instructor(s) taught the virtual patient was a suitable learning style more than a classroom- paper-based cases session.** | **4** | **3** | **2** | **1** | **0** |
| **Overall:** | | | | | | |
| **7** | **I would recommend that the virtual patient could be used in upcoming teaching sessions more than in a classroom- paper-based cases session** | **4** | **3** | **2** | **1** | **0** |
| **8** | **I would rate the quality of the teaching session with the virtual patient in comparison to the classroom- paper-based cases session** | **Excellent**  **4** | **Very good**  **3** | **Good**  **2** | **Average**  **1** | **Poor**  **0** |

**Appendix 4:** Results of tests of validity and reliability of multiple-choice questions of both case 1 and case 2

Case 1 MCQ

| **Question number** | **Facility index** | **Standard deviation** | **Discrimination index** | **Discriminative efficiency** |
| --- | --- | --- | --- | --- |
|  | 73.81% | 44.14% | 33.1% | 45.28% |
|  | 62.70% | 48.55% | 31.6% | 27.33% |
|  | 65.87% | 47.60% | 29.69% | 38.24% |
|  | 63.49% | 48.34% | 29.08% | 36.88% |
|  | 36.51% | 48.34% | 40.81% | 51.70% |
|  | 77.78% | 41.74% | 43.81% | 49.04% |
|  | 51.59% | 50.17% | 40.74% | 49.96% |

Case:2 MCQ

| **Question number** | **Facility index** | **Standard deviation** | **Discrimination index** | **Discriminative efficiency** |
| --- | --- | --- | --- | --- |
|  | 70.79% | 45.73% | 37.35% | 46.78% |
|  | 68.54% | 46.70% | 41.66% | 51.92% |
|  | 65.17% | 47.91% | 45.08% | 55.51% |
|  | 44.94% | 50.03% | 37.16% | 47.57% |
|  | 71.91% | 45.20% | 43.76% | 55.44% |
|  | 73.03% | 44.63% | 54.33% | 69.58% |
|  | 60.67% | 49.12% | 56.51% | 69.19% |
